# Towards a Rational Basis for the Selection of Probiotics to Improve Silkworm Health and Performance

**DOI:** 10.3390/insects16020162

**Published:** 2025-02-04

**Authors:** Siripuk Suraporn, Jisheng Liu, Feifei Ren, Luoluo Wang, Min Feng, Olle Terenius, Luc Swevers

**Affiliations:** 1 Department of Biology, Faculty of Science, Mahasarakham University, Kantarawichai District, Mahasarakham 44150, Thailand; siripuk.s@msu.ac.th; 2 School of Life Sciences, Guangzhou University, Guangzhou 510006, China; jisheng.liu@gzhu.edu.cn; 3 Department of Microbiology, College of Preclinical Medicine, Zunyi Medical University, Zunyi 563006, China; zzff43@outlook.com; 4 National Key Laboratory of Green Pesticide, College of Plant Protection, South China Agricultural University, Guangzhou 510642, China; luoluo.wang@scau.edu.cn; 5 Guangdong Provincial Key Laboratory of Agro-Animal Genomics and Molecular Breeding, College of Animal Science, Regional Sericulture Training Centre for Asia-Pacific, South China Agricultural University, Guangzhou 510642, China; hunanfengmin@scau.edu.cn; 6 Department of Cell and Molecular Biology, Microbiology and Immunology, Uppsala University, Box 596, SE-751 24 Uppsala, Sweden; olle.terenius@icm.uu.se; 7 Insect Molecular Genetics and Biotechnology, Institute of Biosciences and Applications, National Centre for Scientific Research “Demokritos”, 15341 Athens, Greece

**Keywords:** silkworm, *Bombyx mori*, microbiota, silkworm pathogens, microsporidia, BmNPV, probiotics, silk production

## Abstract

Pathogenic infections of silkworms constitute the greatest threat to sericulture. An attractive approach to the improvement in silkworm health and performance comprises the use of probiotics, i.e., microorganisms that confer beneficial properties such as an increased growth rate and resistance against pathogens. While this method has already resulted in promising results, generally, there is a lack of a rational basis for guidance on the selection of probiotics. This review attempts to organize useful information that needs to be considered for the successful application of probiotics: the constitution of the microbiota in silkworms and its origins; the interaction of the major silkworm pathogens with the microbiota; and the microorganisms that have been used so far as silkworm probiotics. Our analysis points to two major issues that seem of vital importance: (1) the absence of a “core microbiota” in silkworms which necessitates continuous supply of beneficial microorganisms according to environmental conditions and (2) the apparent negative impact that some other microorganisms can have on resistance against baculovirus infections. Recent findings have reported the beneficial effects of lactic acid bacteria (*Lactobacillus* sp.) when applied as probiotics in improving silkworm health and performance.

## Simple Summary

Silkworm farming is one of the key economic drivers for farmers in Asia (90% of global silk production). In countries like Thailand, silk production is an important cottage industry that contributes significantly to the economy. Silk is not only used for making silk cloth, but also for other purposes: cosmetics, supplementary food, biological materials, and medical treatments. Waste and frass from silkworm rearing may also be used as fertilizer or for biogas production. However, the greatest challenge in silk production arises from microbial diseases caused by viruses, bacteria, microsporidia, or other fungi. Meanwhile, other microorganisms can serve as beneficial microbes, acting as biological control, and competing with harmful microorganisms. They have the potential to inhibit, or reduce, various pathogens while also being used for feed supplementation to enhance growth in silkworms. Utilizing the potential of silkworm gut microbiota and isolated probiotic bacteria emerges as a strategic approach to promoting silkworm farming within sustainable agroecosystems.

## 1. Introduction: The Importance of Sericulture and Silkworm Rearing

Sericulture refers to the products generated from the silkworm, *Bombyx mori*, which is an insect in the order Lepidoptera, family Bombycidae. The life cycle of *B. mori* is 45–60 days, consisting of the egg, larva, pupa, and adult. Only the larva feeds on mulberry leaves. An artificial diet is considered to be an alternative food for silkworm farming. Commercial silkworm eggs are produced by the crossing of pure paternal lines of silkworm that are most often of Japanese or Chinese origin [1,2]. However, in other countries, native strains were developed that are more tolerant to local conditions and easier to cultivate [3]. For silk production, hybrids (local x local or local x Japanese or Chinese) or poly-hybrids are commonly used.

China, India, Uzbekistan, Vietnam, North Korea, Brazil, and Thailand are the major silk-producing countries (www.inserco.org/en/statistics; URL accessed on 2 January 2025). Although silk represents only a small part of the textile market, its production is spread over more than 60 countries. Sericulture is labor-intensive and employs almost 9 million workers in India and China while 20,000 weaving families live in Thailand (www.inserco.org/en/statistics; URL accessed on 2 January 2025), mostly in the northeastern region or “Isan” [4]. The majority of people in this region grow rice as a staple food. Sericulture can be both the main revenue source and provide extra income for farmers. After rice harvest, farmers continue to rear silkworms as a secondary means to earn extra income. Sericulture therefore plays an important role in securing wages among rural populations while providing raw materials for the silk industry.

The large collection of local silkworm varieties constitutes an important national resource that still awaits largescale validation at the physiological, biochemical, and genetic levels. With the improvements in quality and cost of high-throughput sequencing, efforts have increased to characterize silkworm strains at the genome level to provide insights into the process of artificial selection of silk and other traits [5]. There is a need for the development of molecular markers to distinguish and catalog the different varieties [6,7] while transcriptomic studies can be applied to identify genes that control silk yield [8].

Although silk fabric remains the major outcome of sericulture, nowadays, many value-added products are also associated with silkworm rearing such as cosmetics, supplementary foods, and medical treatments. For instance, silkworm pupae form an excellent source for the growth of the fungus *Cordyceps militaris* and the production of cordycepin, a compound with multiple biologically beneficial activities [9,10,11,12]. Silkworm pupae are used as food with health benefits [13] and are a source of anti-infectious agents [14,15] and cosmetic raw material [16].

As will be more elaborated below, silkworm diseases caused by pathogens are considered major threats in sericulture. The selection of resistant strains is a constant work in progress and advances in biotechnological techniques have opened the possibility for the direct engineering of mechanisms of resistance. However, such approaches can be cumbersome and require specialized equipment and facilities for selection and molecular characterization. For countries in which sericulture remains a cottage industry among local farmers, the use of “probiotics”, i.e., co-habiting microorganisms that provide beneficial effects, represents an attractive approach for the management of silkworms in sericulture. However, relatively little basic information exists about the parameters that have to be considered for this method to become successful. Such information is not restricted to the potential pathogens but should also include the “regular” microbiota as well as the inherent interactions among all co-existing microorganisms in a given silkworm population. For the realization of the potential of the probiotic approach, much more research is needed regarding all pathogen–microbiota interactions that are to be encountered and that will guide the decisions for particular applications.

## 2. Obstacles to Silk Production

The diseases of the silkworm are the greatest threat to sericulture practice [17,18]. An overview of the diverse pathogens that mainly infect silkworm larvae and cause diseases are presented in Table 1.

Several simple practices can be applied to prevent the outbreak of disease, such as disinfection using both physical (sun drying, steam, hot air) and chemical methods (2% formalin, 5% bleaching powder, lime powder, sodium hydroxide solution for inactivation of viral polyhedra). Importantly, silkworms possess an efficient innate immune system to combat infections by pathogens which consists of both cellular (phagocytosis, encapsulation), nodulation, and humoral components (production of antimicrobial peptides, generation of reactive oxygen species, melanization) [24,25]. Of interest is therefore the production of disease-resistant strains of the silkworm.

In the case of BmNPV, multi-omic approaches were applied to elucidate the resistance mechanism in various strains that were bred to oppose viral infection [26,27]. While important information was obtained, in many cases, the exact role of resistance-associated genes could not be determined in detail [28]. Furthermore, it was often observed that breeding for enhanced pathogenic resistance was accompanied by a decline in economically important traits. This could be encountered by the application of transgenic and gene editing techniques used to engineer directly pathogenically resistant traits in economically important strains [29,30]. Accessibility to technologies for the genetic transformation of silkworm strains (e.g., for the overexpression of antiviral proteins or dsRNAs targeting BmNPV genes by RNAi) would require a larger investment in the scientific infrastructure of Asian countries with emerging economies.

Different sensitivities to BmNPV have also been observed for local silkworm strains [31], although the mechanisms have not been clarified. Local BmNPV isolates may represent a monophyletic clade of whose origin can be traced to neighboring countries [32], but how this information can be used for the management of disease resistance remains unexplored.

Although most research has focused on BmNPV infections, resistance mechanisms have been identified at the molecular level for other silkworm diseases as well, such as flacherie [33,34], muscardine [35], and pébrine [36,37]. Transgenic silkworm approaches to combating infection against microsporidia were also reported [38,39].

## 3. The Microbiota of Silkworms

For the better application of probiotics, it is desirable to have a good overview of the microorganisms that are associated with the gut of silkworm larvae. Besides its role of providing innate immunity, the gut microbiota also contributes to resistance against bacterial, fungal, viral, and microsporidian diseases. While oral infection is the main pathway of infection by pathogenic bacteria and viruses, fungal parasites also can penetrate cuticles [40]. However, ecto-microbiota originates from the same environment as gut microbiota [41] and readily becomes assembled from the bacteria of feces [42].

The technique of next-generation sequencing has contributed significantly to our understanding of the hypothesized microbiota of silkworm larvae and has documented changes in their composition according to diet and harmful conditions. The most common molecular method for the exploration of microbial diversity is the sequencing of the 16S rDNA gene, which encodes the smaller ribosomal unit in prokaryotes and has a size of approximately 1.5 kilobase [43]. Results need to be treated with caution, however, since DNA sequencing does not discriminate between dead and live microorganisms or between dormant and growing cells, a limitation which could be addressed by RNA-based sequencing [44]. However, this issue only rarely becomes addressed regarding the sequencing of the silkworm microbiota [45] and the DNA-based method likely overestimates the richness of the microbial community in the silkworm gut.

As in other animals, the gut microbiota of silkworms is considered to be crucially important to enhancing host metabolism and modulating the immune system (but see next section for a more critical view). A considerable number of publications exist regarding the microbiota of (*B. mori*) silkworms that, e.g., make comparisons with the wild silkmoth, *B. mandarina*, and other mulberry-eating polyphagous moth species [45,46] and investigate the impact of artificial diet versus mulberry leaves [47,48,49] as well as mechanisms of resistance against heavy metals, plant toxins, and insecticides [50,51,52]. Unexpectedly, monophagous *B. mori* larvae have a higher microbial diversity than polyphagous mulberry-eating moths, dominated by the phyla Proteobacteria, Firmicutes, Actinobacteria, and Bacteriodetes [45]. *Enterococcus*, *Bacillus*, and *Lactococcus*, representing Gram-positive bacteria belonging to the phylum Firmicutes/Bacillota, were dominant genera [46]. Fungal diversity included mainly species of the phyla Ascomycota and Basidiomycota [45].

Significant differences were observed in microbiota between *B. mori* larvae raised on mulberry leaves and an artificial diet. Two studies observed an increase in microbiota diversity on a diet with mulberry leaves [47,48] while a third report found the highest complexity during feeding with an artificial diet [49]. The artificial diet was found to be easily fermented and increased the amount of lactic acid bacteria in the gut of silkworms [47]. Development of silkworm disease was associated with the dominance of the genera *Enterococcus*, *Lactobacillus*, and *Weissella* (belonging to the order Lactobacillales). Especially *Enterococcus* is considered as a colonizing pathogenic bacterium that is negatively associated with gut acidification and dysbiosis [47]. The transition from artificial diet to mulberry leaves was characterized by increased abundance of *Achromobacter* (Gram-negative, Pseudomonadota/Proteobacteria) and *Rhodococcus* (Gram-positive, Actinomycetota/Actinobacteria), which are associated with the degradation of cellulose and the metabolism of sugars and aromatic compounds, respectively [48]. In the third study [49], the gradual transition from mulberry leaves to artificial diet was characterized by an increase in *Methylobacterium* (Proteobacteria), *Microbacterium* (Actinobacteria), and *Rhodococcus*.

Importantly, the presence of particular bacteria in the gut was associated with xenobiotic metabolism and resistance against harmful chemicals. After the inoculation of germ-free silkworms with the gammaproteobacterium *Stenotrophomonas maltophilia* (isolated from the midgut of 5th instar larvae), an increase in their resistance to the organophosphate insecticide chlorpyrifos was observed, which could be correlated with its capacity to degrade the compound [50]. Feeding of silkworms with leaves of *Cudrania tricuspidata* (Moraceae), for the purpose of production of special high-quality silk, has a large negative effect on larval growth because of the toxicity of prenylated isoflavones. However, supplementation of *Bacillus subtilis* in the diet resulted in an increase in microbial diversity and the detoxification of prenylated isoflavones by glycosylation, thus resolving this growth defect [52]. Feeding on chromium, a heavy metal from the industry, triggered a decline in Proteobacteria concomitant with a rise in species from the order Bacillales, during which the presence of *Weissella paramesenteroides* was noted as indicative of an adaptive response [51].

With respect to silk production, significant increases in cocoon weight and cocoon shell ratio were observed when silkworms were fed mulberry leaves instead of an artificial diet [48]. Although differences in host metabolism must be considered, an altered composition of the microbiota can be assumed to have a significant contribution to silk quality. Some data suggest that the role of the microbiota may not be limited to protection against disease but could be considered as a major factor affecting the quality of the silk that is produced. However, more studies are required to determine the exact role of the microbiota and to uncouple the effects of the microbiota from the food source with which they are associated (see discussion in the next section).

## 4. The Interaction of Silkworm Pathogens with Microbiota

Because many pathogens invade organisms through the gut, the gut microbiota is considered as an important obstacle for infection, either directly, by competition for nutrients, or indirectly, by the stimulation of the host immune response [53,54]. To understand the importance of this interaction in the silkworm, it is necessary to investigate the changes in the microbiota during pathogenic infection. Such research is often accompanied by the determination of the altered metabolome [55], which can generate further insights into the mechanisms by which the microbiota provides assistance to or resistance against invading pathogenic organisms.

### 4.1. Bacterial Microbiota in Silkworms—A Transient Population Dependent on the Environment

Although many studies have documented the presence of bacteria in the gut of lepidopteran larvae (i.e., caterpillars; see above), the existence of microbial symbionts that benefit host growth and development remains ambiguous [56]. This issue has not been analyzed in depth in silkworms but can be inferred from studies with other lepidopterans [56,57]. In silkworms, a few reports have underlined the importance of possibly resident bacteria for the health of the host, e.g., helping the digestion of plant substances [58], while the presence of other bacteria, such as *Staphylococcus*, was associated with pathogenicity [59]. Silkworm larvae, as all other lepidopteran larvae, have a simple digestive tract with high throughput of food and it can be expected that a strong correlation exists between bacteria in the environment (leaves) and the feces [56]. The high microbial variability among individuals contradicts the presence of a silkworm or lepidopteran “core microbiome” that helps digestion, deters pathogens, and provides nutrients, as observed in some other insects such as honeybees, aphids, and termites [56]. Indeed, commercial silkworm artificial diets generally contain antibiotics, which are expected to exacerbate the low microbiota levels. As already discussed above, the microbiota shows major differences between silkworms feeding on mulberry leaves and those feeding on an artificial diet [47,48,49]. The picture that emerges is that silkworm larvae do not need bacteria for robust growth and that the observed microbiota species are mainly diet-derived and transient.

Nevertheless, it is likely that particular transient bacteria in the gut of silkworm larvae can provide protection against toxic substances (as discussed above) as well as pathogenic invaders. The lepidopteran gut, being highly alkaline, is a very harsh environment and it is not always entirely clear to what extent the detection of bacterial 16S rDNA sequences reflects actively functioning versus dead or dormant microbes [45]. Despite the multitude of studies of high-throughput sequencing of 16S rDNA, the presence and interaction of active bacteria in the gut of caterpillars still remains not well understood with respect to their potential benefit for host immunity. Antibiotics that are selective for Gram-positive or -negative bacteria can change the composition of gut microbiota [50]. As already mentioned, *Lactobacillus* bacteria can lower the pH of the gut, which can benefit opportunistic pathogens [47]. However, lowering the pH may also affect the infectivity of microsporidia and baculoviruses (see below). Thus, the success of pathogens seems to be determined by their interaction with other (active) bacteria in the gut and is dependent on their capability to adapt to the harsh gut milieu.

### 4.2. Viruses

During BmNPV infection, occlusion-derived virions are released from the polyhedra in the highly alkaline pH environment that is characteristic of the lepidopteran midgut [60]. Distinct changes in the relative abundance of bacterial families were observed after BmNPV infection, but no significant difference in bacterial diversity was detected [61]. A larger diversity of microbiota was observed in BmNPV-resistant silkworm strains than sensitive strains, which decreased proportionally after BmNPV infection [58,59]. After BmNPV infection, the relative abundances of *Halomonas* (Gammaproteobacteria), *Pseudomonas* (Gammaproteobacteria), *Enterococcus* (Bacilli), and *Aureimonas* (Alphaproteobacteria) in the resistant strain were considerably greater than in the sensitive strain. However, functional enrichment analysis did not indicate a change in the abundances of important functional components of the microbiota in both resistant and sensitive silkworm strains after BmNPV infection [62]. The changes in microbiota following BmNPV infection were found to be strongly dependent on the silkworm strain [63].

Studies with other lepidopteran insects have revealed a strong impact of the microbiota on the resistance against oral baculovirus infection and are mentioned here for its potential relevance to the BmNPV–silkworm infection system. In the cotton bollworm, *Helicoverpa armigera*, elimination of the microbiota by treatment with antibiotics significantly increased resistance to *H. armigera* nucleopolyhedrovirus (HaNPV) [64]. More specifically, the presence of microbiota decreased the expression of prophenoloxidase (PPO), which has antiviral activity. In addition, while the bacterial diversity decreased during HaNPV infection, the bacterial load increased (especially *Enterococcus*), resulting in higher expression of antimicrobial peptides (AMPs) by the host [64]. An increase in the bacterial load in the gut was also observed during infection of *Spodoptera exigua* larvae with *S. exigua* multiple nucleopolyhedrovirus (SeMNPV), which was associated with a decrease in the expression of immune genes [65]. Also, in this infection model, the absence of microbiota strongly increased the resistance of the larvae against SeMNPV infection. Melanization is also induced in silkworm larvae and occurs at higher levels in a susceptible strain than in a resistant one [66]. Interestingly, resistant silkworm strains can block BmNPV infection in the presence of an inhibitor of melanization, indicating additional mechanisms of defense [66]. In the PO cascade, reactive oxygen species typically are also produced that can have antiviral activity [67]. Given the evidence in other Lepidoptera, the negative regulation of the PO system by particular microbiota needs to be kept in mind when probiotics are applied to particular silkworm strains. The importance of the melanization reaction in anti-BmNPV defense requires further investigation.

While the above observations suggest that (some) microbiota can decrease their resistance against BmNPV, it was recently observed that the feeding of lactic acid bacteria (*Lactobacillus acidophilus*) could prevent BmNPV infection of silkworms [68]. Possible mechanisms include the acidification of the midgut and competition for adhesion to the midgut epithelium. Future experiments should clarify the immune response mechanisms induced by *Lactobacillus* and to what extent these can differ from those induced by other microbiota.

In contrast to BmNPV, silkworm gut infections with *B. mori* Cypovirus (formerly known as cytoplasmic polyhedrosis virus, BmCPV) (*Reoviridae*) are much less virulent such that often persistent infections are established [69]. BmCPV specifically infects the epithelium of the midgut, thereby affecting digestive and absorptive functions [70]. Bacterial diversity declined after BmCPV infection and bacteria of the genus *Enterococcus* became particularly predominant [71]. BmCPV infection was reported to induce the expression of cecropin AMPs [69], which may have an impact on the silkworm microbiota.

Similar observations were made during BmDNV infection, which resulted in a decrease in bacterial diversity with *Enterococcus* as the dominant species, concomitant with an activation of immune genes [72].

### 4.3. Fungi

In the formation of micro-ecological networks, fungi and bacteria are the most active participants encompassing a variety of both positive and negative interactions [73]. The feeding of antibacterial or antifungal antibiotics significantly altered the gut microbiota of silkworms [74]. However, loss of fungi decreased the bacterial diversity and richness while the fungal diversity index increased following treatment with antibacterial penicillin and streptomycin, illustrating the complex relationship between the two types of microorganisms.

Pathogenic fungi are known to secrete antimicrobial peptides and antibiotics to facilitate infection and growth [75,76]. Similarly, bacterial species of the genus *Bacillus* have been used to combat fungal pathogens [77]. With respect to silkworms, the impact of bacteria in antifungal defense was illustrated by the observation that the acquisition of *Mammaliicoccus sciuri* (class Bacilli, family Staphylococcaceae), which was part of the phyllosphere of mulberry leaves, could prevent the germination of entomopathogenic fungi such as *Beauveria* and *Aspergillus* [42]. Protection by the bacteria, which became part of the cuticular ecto-microbiota, was dependent on the secretion of a chitinolytic lysozyme that damaged the fungal cell walls [42].

### 4.4. Microsporidia

Together with grasserie, caused by BmNPV infection, pébrine is considered the most destructive disease for sericulture practice [32,36]. *Nosema bombycis* was described more than 150 years ago as the first microsporidium to cause pébrine disease in silkworm larvae [78]. In recent years, it has become apparent that microsporidia can have a large effect on the microbiome and that other microorganisms can prevent infection by microsporidia [79].

Microsporidia have a sophisticated mechanism of host invasion during which a polar tube becomes uncoiled from the spores for deposition of the sporoplasm in the cellular cytoplasm [80]. The parasitic life cycle continues by a proliferative stage called meront and completes by differentiation of mature spores. In silkworms, microsporidia can be transmitted both horizontally and vertically [81]. The *N. bombycis* genome was sequenced [82] and the *N. bombycis*–*B. mori* parasite–host pair has emerged as an important model for the study of the microsporidian life cycle [39,80]. Research has resulted in the identification of microsporidian genes and proteins that can be targeted by specific antibodies or by RNAi for the inhibition of the infection cycle [83,84,85]. Significant advances have also been made with respect to the characterization of the immune response against *N. bombycis* in silkworms [36,86,87,88,89]. More robust and sophisticated methods for the detection of microsporidia are continuously being developed [90,91,92,93] and several chemical drugs are available to treat pébrine disease [94,95].

*N. bombycis* is well adapted to infect the alkaline midgut of silkworm larvae [94,96]. After infection of the midgut epithelium, it can spread to internal tissues such as the muscles, Malpighian tubules, and gonads [97]. Infection by *N. bombycis* resulted in a decrease in the bacterial diversity and richness in the silkworm gut while the total bacterial load increased significantly [98]. More specifically, a positive correlation could be observed between the abundance of *N. bombycis* and *Enterococcus* bacteria. Interestingly, co-feeding of the bacterial strain *Enterococcus faecalis* LX10 resulted in significant reduction in microsporidium invasion and gut epithelial cell damage [98]. Thus, in the presence of the *Enterococcus* strain, the virulence of *N. bombycis* was significantly reduced, with as possible mechanisms the reduction in the pH in the gut and the production of enterococcin EntV, which has AMP activity against *N. bombycis* [98].

Interactions between baculovirus and *Nosema* infection were observed in another lepidopteran insect, the gypsy moth *Lymantria dispar* [99]. Larvae that were pre-infected with *Nosema* showed an increased sensitivity to LdNPV infection and the time to death was significantly shorter. In the presence of *Nosema*, the number of polyhedra was strongly diminished, leading to the proposal that a greater diversity of pathogens, such as microsporidia, could dampen outbreaks of baculovirus and stabilize moth populations [98].

The exposition above makes clear that both positive and negative interactions occur among the microorganisms associated with silkworms and significant physiological and immunological changes therefore can be expected following changes in the environment, which include the introduction of probiotics for improvement in silkworm characteristics.

## 5. Using Microorganisms to Promote Silk Production

Probiotics are defined as “live microorganisms that, when administered in adequate amounts, confer a health benefit on the host” [100]. While apparently conceived to promote human health, the concept has expanded to improve livestock farming and aquaculture [101]. Benefits of probiotics include the modulation of the immune response, tolerance against food antigens, competition with pathogens for adhesion and nutrients, enhancement in intestinal barrier function, production of bacteriocins, scavenging of superoxide radicals, and modification of toxins (WGO Global Guideline Probiotics and prebiotics—www.worldgastroenterology.org; URL accessed on 10 December 2024). Probiotics are also increasingly used to maintain the health of economically important insects for food production [102] and in apiculture [103,104] and sericulture [105,106].

Microorganisms with probiotic activity in mammals (*Bifidobacteria*, *Lactobacilli*, yeast, *Staphylococcus*, and *Bacillus* species) have also been reported to improve silkworm characteristics such as the growth rate and cocoon weight and size [107,108,109,110,111,112,113]. Such improvements could also be achieved with indigenous or endophytic probiotic bacteria [114,115]. Important characteristics include the capacity of the bacteria to efficiently digest major dietary ingredients such as pectin, starch, cellulose, xylan, and lipids from the host plant [58,116]. Other applications include the use of probiotics to inhibit infections by pathogens of viral [68], bacterial [117,118,119], fungal [120], and microsporidian [121,122,123] origin. An overview of experiments on the successful application of probiotics on the silkworm *B. mori* is presented in Table 2. A recent review also summarized the effects of probiotic supplementation in *B. mori* [124].

In a number of recent studies, multi-omic approaches were used to analyze the effects of specific probiotics at the molecular level, which revealed possible mechanisms by which probiotics could increase the growth and silk production of silkworms [125,126,127,128]. Treatment with *Lactobacillus reuteri* resulted in an increase in mRNA levels of mitogen-activated protein kinase (MAPK) and phosphatidylinositide 3-kinase (PI3K) as well as the silk gland-specific transcription factor SGF1 in silkworms [127]. Furthermore, levels of ecdysone were elevated. Also changes in the microbiota were observed and feeding with *L. reuteri* resulted in greater bacterial diversity [127]. Application of a *Bacillus subtilis* probiotic resulted in higher expression of AMPs and antioxidant enzymes [125]. An increase in the levels of vitamins in the hemolymph was also detected [125]. The mechanism by which *Pediococcus pentosaceus* (Lactobacillales) stimulated larval growth and resistance against the pathogenic bacterium *Enterococcus mundtii* (isolated from larvae with flacherie disease) was also investigated [128]. Gut digestive enzymatic activity was increased together with the levels of the AMP attacin and the antioxidant capacity. It was observed that *P. pentosaceus* could efficiently colonize the gut, which resulted in changes in the microbiota. Changes in metabolites were also observed, most notably an increase in glycine, which is considered especially important for silk synthesis [129]. Although implicated in flacherie disease, *Enterococcus mundtii* was also reported to have beneficial effects for silkworms such as a decrease in microbial dysbiosis in the gut, most notably a negative effect on *Staphylococcus* abundance, possibly as a consequence of the induction of AMP genes [130]. In the latter study, *E. mundtii* did not affect silkworm metabolism or the integrity of the gut epithelium [130].

The available data therefore indicate that probiotics can be used successfully in sericulture to maintain the health of silkworms. Caution is required, however, as illustrated by the observation that the absence of microbiota can increase resistance against baculovirus infection, due to the higher expression of the antiviral PPO system [64,65]. Nevertheless, the feeding of *Lactobacillus* not only improved silkworm growth and cocoon quality parameters but also provided protection against BmNPV (and microsporidia) [68,122]. For the application of probiotics, it is necessary to investigate in more detail the innate immune response for the elucidation of the resistance mechanisms that could provide protection against diverse pathogens.

## 6. Conclusions: Management of Microorganisms and Their Application

**Figure 1:**
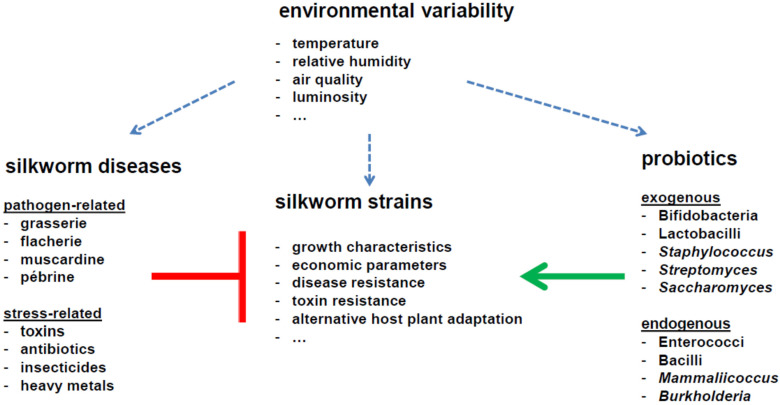
The application of probiotics to improve sericultural practices.

This review was written to provide a basis for the rational application of probiotics to improve silkworm maintenance in sericulture. In countries with limited scientific infrastructure, the use of probiotics can provide an environmentally friendly and a relatively cheap solution for the management of different silkworm strain populations. Encouragingly, a significant body of work has shown the positive effect of different microorganisms in improving the health of silkworms and the economic parameters of cocoon silk. However, much more research effort is necessary for the systematic application of probiotics against specific threats that are encountered (Figure 1). First of all, more work is needed to identify additional beneficial microorganisms in the natural environment [131]. Candidate microorganisms can be isolated from the midgut tissue of silkworm strains by their differential abundance in healthy and diseased silkworms. Another issue relates to the stability of the association of microorganisms with silkworms, since silkworms may not be able to maintain a persistent microbiota—in such case, probiotics may need to be continuously applied to maintain beneficial effects. A major question, which hitherto has largely been neglected, concerns the differential activation of an immune response by microorganisms. Recently, the interaction of an immune response with the regulation of growth in silkworms has become apparent [132,133,134,135]. Many mechanisms behind antiviral response especially remain to be uncovered [136,137,138,139] and pathogenic recognition receptors that are involved in antibacterial defense may play an opposite (permissive) role with respect to viral infection [140].

While probiotics such as *Lactobacillus* bacteria can provide benefits for silkworm maintenance, the mechanisms remain obscure at this point. There is a need for the clarification of the immune response pathways induced by lactic bacteria and how these can differ from other bacteria (microbiota). Thus, probiotics need to be screened to optimize their application potential. It is clear that much more work is needed to clarify the interactions among beneficial and pathogenic microorganisms for the optimal and flexible application of probiotics in sericulture.

## Figures and Tables

**Table 1:** Main silkworm pathogens and their diseases.

Disease	Causative Pathogens	References
Grasserie	Viruses *Bombyx mori* Nucleopolyhedrovirus (BmNPV) (*Baculoviridae*)	[17,18,19,20]
Flacherie	Viruses *Bombyx mori* Infectious flacherie virus (BmIFV) (*Iflaviridae*) *Bombyx mori* Densonucleosis virus (BmDNV) (*Bidnaviridae*) *Bombyx mori* Cypovirus (BmCPV) (*Reoviridae*) Bacteria *Streptococcus* sp. *Staphylococcus* sp. *Bacillus thuringiensis* *Serratia marcescens* Combination of viruses and bacteria	[17,18,21,22]
Muscardine	Fungi *Beauveria bassiana* (white muscardine)	[17,18,19,23]
Pébrine	Microsporidia *Nosema bombycis*	[17,18,19,22]

**Table 2:** List of microorganisms that have been used as probiotics in the domesticated silkworm, *Bombyx mori*.

Probiotics	Impacts	Reference
*Bacillus amyloliquefaciens* *Bacillus cereus*	increase cocoon weight increase sericin and fibroin content	[113]
*Bacillus licheniformis* *Bacillus niabensis*	increase larval survival increase larval weight increase pupal weight increase cocoon weight increase cocoon shell ratio	[111]
*Bacillis subtilis*	increase larval weight increase antioxidant properties increase antimicrobial peptide expression increase vitamin levels	[125]
*Bifidobacterium*	improve production of raw silk	[110]
*Burkholderia cepacia*	increase protease activity in midgut	[114]
*Lactobacillus plantarum*	increase larval weight increase cocoon shell weight increase pupation rate	[107]
Lact-Act (commercial probiotic)	increase survival against *Bacillus thuringiensis* and *Staphylococcus aureus*	[117]
*Lactobacillus acidophilus*	increase survival increase larval weight increase pupation ratio increase cocoon weight increase cocoon-shell ratio	[109]
*Lactobacillus casei* *Lactobacillus plantarum*	increase cocoon weight increase sericin and fibroin content	[113]
*Lactococcus lactis* yoghurt	protection against *Pseudomonas aeruginosa* protection against *Staphylococcus aureus*	[126]
*Lactobacillus paraplantarum*	protection against *Pseudomonas aeruginosa*	[119]
*Lactobacillus casei*	increase survival against *Nosema* increase larval weight (*Nosema* infected) increase pupation ratio (*Nosema* infected)	[122]
*Lactobacillus rhamnosus*	increase larval survival increase larval weight increase cocoon weight increase pupation rate	[123]
*Lactobacillus acidophilus*	increase survival against BmNPV increase larval weight (BmNPV infected) increase pupation ratio (BmNPV infected) increase cocoon weight (BmNPV infected)	[68]
*Lactobacillus reuteri*	increase larval weight increase cocoon weight increase cocoon shell ratio increase growth factor signaling	[127]
*Pediococcus pentosaceus*	increase cocoon shell weight increase cocoon shell ratio increase digestive activity gut increase antimicrobial peptides increase antioxidant enzymes	[128]
*Saccharomyces cerevisiae*	increase pupal weight increase cocoon weight increase cocoon shell ratio increase silk filament length increase amylase and invertase activity in midgut	[108]
*Staphylococcus gallinarum* *Staphylococcus arlettae*	increase larval survival increase larval weight increase pupal weight increase cocoon weight increase cocoon shell ratio increase silk filament length and weight	[112]
*Trichoderma harzianumas*	increase pupal weight increase cocoon weight increase cocoon shell ratio protection against *Metarhizium anisopliae*	[120]

## References

[1] Giora D., Marchetti G., Cappellozza S., Assirelli A., Saviane A., Sartori L., Marinello F. (2022). Bibliometric analysis of trends in mulberry and silkworm research on the production of silk and its by-products. Insects.

[2] Cappellozza S., Casartelli M., Sandrelli F., Saviane A., Tettamanti G. (2022). Silkworm and silk: Traditional and innovative applications. Insects.

[3] Nasirillaev B., Rajabov N., Abdukadirov M., Fozilova K. (2023). History and development prospects of silk farming in Uzbekistan. E3S Web Conf..

[4] Sumranpath K., Aungsuratana A., Auttathom T., Poramacom N. (2015). Existing condition of commercial sericulture production in Northeastern Thailand. Kasetsart J. Soc. Sci..

[5] Tong X., Han M.J., Lu K., Tai S., Liang S., Liu Y., Hu H., Shen J., Long A., Zhan C. (2022). High-resolution silkworm pan-genome provides genetic insights into artificial selection and ecological adaptation. Nat. Commun..

[6] Kim K.Y., Kang P.D., Lee K.G., Oh H.K., Kim M.J., Kim K.-H., Park S.W., Lee S.J., Jin B.R., Kim I. (2010). Microsatellite analysis of the silkworm strains (Bombyx mori): High variability and potential markers for strain identification. Genes Genom..

[7] Park J.S., Kim M.J., Kim S.-W., Kim K.-Y., Kim S.-R., Kim I. (2022). Molecular identification of the strains of the domestic silkworm, *Bombyx mori* (Lepidoptera: Bombycidae), which are endemic to Korea, based on single nucleotide polymorphisms in mitochondrial genome sequences. J. Asia Pac. Entomol..

[8] Luan Y., Zuo W., Li C., Gao R., Zhang H., Tong X., Han M., Hu H., Lu C., Dai F. (2018). Identification of genes that control silk yield by RNA sequencing analysis of silkworm (*Bombyx mori*) strains of variable silk yield. Int. J. Mol. Sci..

[9] Suraporn S. (2015). Growth of *Cordyceps militaris* on Plant Substrates Supplemented with Pupal Powder of Thai Variety of the Silkworm, *Bombyx mori*. Int. J. Wild Silkmoth Silk.

[10] Suraporn S., Raman C. (2016). Mycelium production of *Cordyceps militaris* Thai strain on Thai rice varieties mixed with yolk and pupal powder of Thai silkworm, *Bombyx mori*. Sericologia.

[11] Suraporn S., Siriwattanametanon W. (2019). Growth of *Cordyceps* spp. on the pupae of Thai silkworm, *Bombyx mori* Nanglaix108. J. Kamphaengsean Acad. Kasetsart Univ. Thail..

[12] Li Y.-T., Yao H.-T., Huang Z.-L., Gong L.-C., Herman R.A., Wu F.-A., Wang J. (2024). Silkworm pupae globulin promotes *Cordyceps militaris* fermentation: Regulation of metabolic pathways enhances cordycepin synthesis and extends the synthesis phase. Food Biosci..

[13] Zhou Y., Zhou S., Duan H., Wang J., Yan W. (2022). Silkworm pupae: A functional food with health benefits for humans. Foods.

[14] Panthee S., Paudel A., Hamamoto H., Sekimizu K. (2017). Advantages of the silkworm as an animal model for developing novel antimicrobial agents. Front. Microbiol..

[15] Hamamoto H., Horie R., Sekimizu K. (2019). Pharmacokinetics of anti-infectious reagents in silkworms. Sci. Rep..

[16] Heryati Y., Sarwono K.A., Riendriasari S.D., Andadari L., Agustarini R., Arung E.T., Fatriasari W., Kusuma I.W., Kuspradini H., Shimizu K., Kim Y.-u., Azelee N.I.W., Edis Z. (2024). Silkworm for Cosmetic Application. Biomass-Based Cosmetics.

[17] Watanabe H. (2002). Genetic resistance of the silkworm, *Bombyx mori* to viral diseases. Curr. Sci..

[18] Nuraeni S. (2017). Gaps in the thread: Disease, production, and opportunity in the failing silk industry of South Sulawesi. Forest Soc..

[19] Guo-Ping K., Xi-Jie G. (2011). Overview of silkworm pathology in China. Afr. J. Biotechnol..

[20] Gani M., Chouhan S., Babulal, Gupta R.K., Khan G., Bharath Kumar N., Saini P., Ghosh M.K. (2018). *Bombyx mori* nucleopolyhedrovirus (BmNPV): Its impact on silkworm rearing and management strategies. J. Biol. Control.

[21] Manimegalai S. (2009). Bacterial pathogens of mulberry silkworm, *Bombyx mori* L. and their management strategies. Sericologia.

[22] Bhat S., Bashir I., Kamili A. (2009). Microsporidiosis of silkworm, *Bombyx mori* L. (Lepidoptera-Bombycidae): A review. Afric. J. Agric. Res..

[23] Nirupama R. (2014). Fungal disease of white muscardine in silkworm, *Bombyx mori* L.. Munis Entomol. Zool..

[24] Chandana M., Bhaskar R.N., Prasanna Kumar M.K. (2020). Isolation, characterization and molecular identification of bacterial isolates associated with *Bombyx mori* Cytoplasmic Polyhedrosis Virus (*Bm*CPV) infected silkworm midgut. Int. J. Curr. Microbiol. App. Sci..

[25] Sharma A., Sharma P., Thakur J., Murali S., Bali K. (2020). Viral diseases of mulberry silkworm, *Bombyx mori* L.—A review. J. Pharmacogn. Phytochem..

[26] Bao Y.Y., Tang X.D., Lv Z.Y., Wang X.Y., Tian C.H., Xu Y.P., Zhang C.X. (2009). Gene expression profiling of resistant and susceptible *Bombyx mori* strains reveals nucleopolyhedrovirus-associated variations in host gene transcript levels. Genomics.

[27] Yu L., Cao Y., Ge S., Xu A., Qian H., Li G. (2022). Identification of key genes involved in resistance to early stage of BmNPV infection in silkworms. Viruses.

[28] Jiang L., Goldsmith M.R., Xia Q. (2021). Advances in the arms race between silkworm and baculovirus. Front. Immunol..

[29] Jiang L., Xia Q. (2014). The progress and future of enhancing antiviral capacity by transgenic technology in the silkworm *Bombyx mori*. Insect Biochem. Mol. Biol..

[30] Hu Z., Zhu F., Chen K. (2023). The mechanisms of silkworm resistance to the baculovirus and antiviral breeding. Annu. Rev. Entomol..

[31] Suraporn S., Terenius O. (2020). Sensitivity of polyvoltine Thai strains of *Bombyx mori* to a BmNPV isolate From Mahasarakham. J. Insect Sci..

[32] Wennmann J.T., Senger S., Ruoff B., Jehle J.A., Suraporn S. (2024). Distribution and genetic diversity of *Bombyx mori* nucleopolyhedrovirus in mass-reared silkworms in Thailand. J. Invertebr. Pathol..

[33] Mondal R., Dam P., Chakraborty J., Shaw S., Pradhan S., Das S., Nesa J., Meena K., Ghati A., Chaudhuri S.D. (2024). Genomic dataset of a multiple-drug resistant *Pseudomonas* sp. strain RAC1 isolated from a flacherie infected Nistari race of *Bombyx mori* L.. Data Brief.

[34] Murthy G.N., Ponnuvel K.M., Awasthi A.K., Rao C.G., Chandrasekhar Sagar B.K. (2014). The Indian isolate of Densovirus-2—Impact of infection and mechanism of resistance in *Bombyx mori* L.. J. Invertebr. Pathol..

[35] Hou C., Qin G., Liu T., Geng T., Gao K., Pan Z., Qian H., Guo X. (2014). Transcriptome analysis of silkworm, *Bombyx mori*, during early response to *Beauveria bassiana* challenges. PLoS ONE.

[36] Li Z., Wang Y., Wang L., Zhou Z. (2018). Molecular and biochemical responses in the midgut of the silkworm, *Bombyx mori*, infected with *Nosema bombycis*. Parasit. Vectors.

[37] Nuraeni S., Sadapotto A. (2019). Testing of two microsporidia isolates towards breeds of silkworm resistance. IOP Conf. Ser. Earth Environ. Sci..

[38] Dong Z., Long J., Huang L., Hu Z., Chen P., Hu N., Zheng N., Huang X., Lu C., Pan M. (2019). Construction and application of an HSP70 promoter-inducible genome editing system in transgenic silkworm to induce resistance to *Nosema bombycis*. Appl. Microbiol. Biotechnol..

[39] Dong Z., Zheng N., Hu C., Huang X., Chen P., Wu Q., Deng B., Lu C., Pan M. (2021). Genetic bioengineering of overexpressed guanylate binding protein family BmAtlastin-n enhances silkworm resistance to *Nosema bombycis*. Int. J. Biol. Macromol..

[40] Hong S., Sun Y., Sun D., Wang C.S. (2022). Microbiome assembly on *Drosophila* body surfaces benefits the flies to combat fungal infections. iScience.

[41] Hong S., Shang J., Sun Y., Tang G., Wang C.S. (2023). Fungal infection of insects: Molecular insights and prospects. Trends Microbiol..

[42] Zhao P., Hong S., Li Y., Chen H., Gao H., Wang C. (2024). From phyllosphere to insect cuticles: Silkworms gather antifungal bacteria from mulberry leaves to battle fungal parasite attacks. Microbiome.

[43] Ponnusamy M., Karthikeyan C.V., Ramanathan B., Tripathi V., Kumar P., Tripathi P., Kishore A., Kamle M. (2019). Meta-omics in detection of silkworm gut microbiome diversity. Microbial Genomics in Sustainable Agroecosystems.

[44] Li R., Tun H.M., Jahan M., Zhang Z., Kumar A., Dilantha Fernando W.G., Farenhorst A., Khafipour E. (2017). Comparison of DNA-, PMA-, and RNA-based 16S rRNA Illumina sequencing for detection of live bacteria in water. Sci. Rep..

[45] Chen B., Du K., Sun C., Vimalanathan A., Liang X., Li Y., Wang B., Lu X., Li L., Shao Y. (2018). Gut bacterial and fungal communities of the domesticated silkworm (*Bombyx mori*) and wild mulberry-feeding relatives. ISME J..

[46] Kumar D., Sun Z., Cao G., Xue R., Hu X., Gong C. (2019). Study of gut bacterial diversity of *Bombyx mandarina* and *Bombyx mori* through 16S rRNA gene sequencing. J. Asia Pac. Entomol..

[47] Qin L., Qi J., Shen G., Qin D., Wu J., Song Y., Cao Y., Zhao P., Xia Q. (2022). Effects of microbial transfer during food-gut-feces circulation on the health of *Bombyx mori*. Microbiol. Spectr..

[48] Wang G., Ding X., Yang J., Ma L., Sun X., Zhu R., Lu R., Xiao Z., Xing Z., Liu J. (2024). Effects of habitual dietary change on the gut microbiota and health of silkworms. Int. J. Mol. Sci..

[49] Xin L., Chen Y., Rong W., Qin Y., Li X., Guan D. (2024). Gut microbiota analysis in silkworms (*Bombyx mori*) provides insights into identifying key bacterials for inclusion in artificial diet formulations. Animals.

[50] Chen B., Zhang N., Xie S., Zhang X., He J., Muhammad A., Sun C., Lu X., Shao Y. (2020). Gut bacteria of the silkworm *Bombyx mori* facilitate host resistance against the toxic effects of organophosphate insecticides. Environ. Int..

[51] Chen Y.-Z., Rong W.-T., Qin Y.-C., Lu L.-Y., Liu J., Li M.-J., Xin L., Li X.-D., Guan D.-L. (2023). Integrative analysis of microbiota and metabolomics in chromium-exposed silkworm (*Bombyx mori*) midguts based on 16S rDNA sequencing and LC/MS metabolomics. Front. Microbiol..

[52] Yuan S., Sun Y., Chang W., Zhang J., Sang J., Zhao J., Song M., Qiao Y., Zhang C., Zhu M. (2023). The silkworm (*Bombyx mori*) gut microbiota is involved in metabolic detoxification by glucosylation of plant toxins. Commun. Biol..

[53] Bäumler A.J., Sperandio V. (2016). Interactions between the microbiota and pathogenic bacteria in the gut. Nature.

[54] McKenney E.S., Kendall M.M. (2016). Microbiota and pathogen ’pas de deux’: Setting up and breaking down barriers to intestinal infection. Pathog. Dis..

[55] Wang L., Smagghe G., Swevers L., Lu Y. (2024). Metabolomics-based approaches in unraveling virus infections in insects: Revealing unknown hidden interactions. Entomol. General..

[56] Hammer T.J., Janzen D.H., Hallwachs W., Jaffe S.P., Fierer N. (2017). Caterpillars lack a resident gut microbiome. Proc. Natl. Acad. Sci. USA.

[57] Paniagua Voirol L.R., Frago E., Kaltenpoth M., Hilker M., Fatouros N.E. (2018). Bacterial symbionts in Lepidoptera: Their diversity, transmission, and impact on the host. Front. Microbiol..

[58] Anand A.A.P., Vennison S.J., Sankar S.G., Prabhu D.I., Vasan P.T., Raghuraman T., Geoffrey C.J., Vendan S.E. (2010). Isolation and characterization of bacteria from the gut of *Bombyx mori* that degrade cellulose, xylan, pectin and starch and their impact on digestion. J. Insect Sci..

[59] Haloi K., Kalita M.K., Nath R., Devi D. (2016). Characterization and pathogenicity assessment of gut-associated microbes of muga silkworm *Antheraea assamensis* Helfer (Lepidoptera: Saturniidae). J. Invertebr. Pathol..

[60] Wang M., Hu Z. (2019). Cross-talking between baculoviruses and host insects towards a successful infection. Philos. Trans. R. Soc. Lond. B Biol. Sci..

[61] Shi X., Zhang Y., Zhu T., Li N., Sun S., Zhu M., Pan J., Shen Z., Hu X., Zhang X. (2021). Response to Bombyx mori nucleopolyhedrovirus infection in silkworm: Gut metabolites and microbiota. Dev. Comp. Immunol..

[62] Yang X., Liu P., Yu H., Ling M., Ma M., Wang Q., Tang X., Shen Z., Zhang Y. (2024). Comparative analysis of the intestinal flora of BmNPV-resistant and BmNPV-sensitive silkworm varieties. Microb. Pathog..

[63] Liu S.H., Zhang Y., Guo Z.X., Ayaz S., Wang Y.X., Huang Z.H., Cao H.H., Xu J.P. (2024). Effects of baculovirus infection on intestinal microflora of BmNPV resistant and susceptible strain silkworm. J. Econ. Entomol..

[64] Yuan C., Xing L., Wang M., Hu Z., Zou Z. (2021). Microbiota modulates gut immunity and promotes baculovirus infection in *Helicoverpa armigera*. Insect Sci..

[65] Jakubowska A.K., Vogel H., Herrero S. (2013). Increase in gut microbiota after immune suppression in baculovirus-infected larvae. PLoS Pathog..

[66] Chen T.T., Hu N., Tan L.R., Xiao Q., Dong Z.Q., Chen P., Xu A.Y., Pan M.H., Lu C. (2019). Resistant silkworm strain block viral infection independent of melanization. Pestic Biochem. Physiol..

[67] Liu Y.X., Yang J.Y., Sun J.L., Wang A.C., Wang X.Y., Zhu L.B., Cao H.H., Huang Z.H., Liu S.H., Xu J.P. (2023). Reactive oxygen species-mediated phosphorylation of JNK is involved in the regulation of BmFerHCH on Bombyx mori nucleopolyhedrovirus proliferation. Int. J. Biol. Macromol..

[68] Suraporn S., Suthikhum V., Terenius O. (2024). The mortality of *Bombyx mori* larvae challenged by BmNPV is reduced when supplemented with *Lactobacillus acidophilus* bacteria. BMC Res. Notes.

[69] Kolliopoulou A., Van Nieuwerburgh F., Stravopodis D.J., Deforce D., Swevers L., Smagghe G. (2015). Transcriptome analysis of Bombyx larval midgut during persistent and pathogenic cytoplasmic polyhedrosis virus infection. PLoS ONE.

[70] Swevers L., Feng M., Ren F., Sun J. (2020). Antiviral defense against Cypovirus 1 (*Reoviridae*) infection in the silkworm, *Bombyx mori*. Arch. Insect Biochem. Physiol..

[71] Sun Z., Lu Y., Zhang H., Kumar D., Liu B., Gong Y., Zhu M., Zhu L., Liang Z., Kuang S. (2016). Effects of BmCPV infection on silkworm *Bombyx mori* intestinal bacteria. PLoS ONE.

[72] Kumar D., Sun Z., Cao G., Xue R., Hu X., Gong C. (2019). *Bombyx mori* bidensovirus infection alters the intestinal microflora of fifth instar silkworm (*Bombyx mori*) larvae. J. Invertebr. Pathol..

[73] Gao X., Huynh B.-T., Guillemot D., Glaser P., Opatowski L. (2018). Inference of significant microbial interactions from longitudinal metagenomics data. Front. Microbiol..

[74] Li C., Xu S., Xiang C., Xu S., Zhou Q., Zhang J. (2022). The gut microbiota of silkworm are altered by antibiotic exposure. FEBS Open Bio.

[75] Sun Y.L., Chen B., Li X.L., Yin Y., Wang C.S. (2022). Orchestrated biosynthesis of the secondary metabolite cocktails enables the producing fungus to combat diverse bacteria. mBio.

[76] Hong S., Sun Y.L., Chen H.M., Wang C.S. (2023). Suppression of the insect cuticular microbiomes by a fungal defensin to facilitate parasite infection. ISME J..

[77] Kaspar F., Neubauer P., Gimpel M. (2019). Bioactive secondary metabolites from *Bacillus subtilis*: A comprehensive review. J. Nat. Prod..

[78] Franzen C. (2008). Microsporidia: A review of 150 years of research. TOPARAJ.

[79] Tersigni J., Tamim El Jarkass H., James E.B., Reinke A.W. (2024). Interactions between microsporidia and other members of the microbiome. J. Eukaryot. Microbiol..

[80] Tamim El Jarkass H., Reinke A.W. (2020). The ins and outs of host-microsporidia interactions during invasion, proliferation and exit. Cell. Microbiol..

[81] Hukuhara T. (2018). The epizootiology of pebrine, one of the great scourges of sericulture. J. Biochem. Biotech..

[82] Pan G., Xu J., Li T., Xia Q., Liu S.L., Zhang G., Li S., Li C., Liu H., Yang L. (2013). Comparative genomics of parasitic silkworm microsporidia reveal an association between genome expansion and host adaptation. BMC Genom..

[83] Zhang F., Lu X., Kumar V.S., Zhu H., Chen H., Chen Z., Hong J. (2007). Effects of a novel anti-exospore monoclonal antibody on microsporidial *Nosema bombycis* germination and reproduction in vitro. Parasitology.

[84] Huang Y., Chen J., Sun B., Zheng R., Li B., Li Z., Tan Y., Wei J., Pan G., Li C. (2018). Engineered resistance to *Nosema bombycis* by in vitro expression of a single-chain antibody in Sf9-III cells. PLoS ONE.

[85] He Q., Vossbrinck C.R., Yang Q., Meng X.Z., Luo J., Pan G.Q., Zhou Z.Y., Li T. (2019). Evolutionary and functional studies on microsporidian ATPbinding cassettes: Insights into the adaptation of microsporidia to obligated intracellular parasitism. Infect. Genet. Evol..

[86] Ma Z., Li C., Pan G., Li Z., Han B., Xu J., Lan X., Chen J., Yang D., Chen Q. (2013). Genome-wide transcriptional response of silkworm (*Bombyx mori*) to infection by the microsporidian *Nosema bombycis*. PLoS ONE.

[87] Bao J., Liu L., An Y., Ran M., Ni W., Chen J., Wei J., Li T., Pan G., Zhou Z. (2019). *Nosema bombycis* suppresses host hemolymph melanization through secreted serpin 6 inhibiting the prophenoloxidase activation cascade. J. Invertebr. Pathol..

[88] Ni W., Bao J., Mo B., Liu L., Li T., Pan G., Chen J., Zhou Z. (2020). Hemocytin facilitates host immune responses against *Nosema bombycis*. Dev. Comp. Immunol..

[89] Hua X., Xu W., Ma S., Xia Q. (2021). STING-dependent autophagy suppresses *Nosema bombycis* infection in silkworms, *Bombyx mori*. Dev. Comp. Immunol..

[90] Yan W., Shen Z., Tang X., Xu L., Li Q., Yue Y., Xiao S., Fu X. (2014). Detection of *Nosema bombycis* by FTA cards and loop-mediated isothermal amplification (LAMP). Curr. Microbiol..

[91] Kampliw S., Monthatong M. (2019). Loop mediated isothermal amplification (LAMP) for *Nosema bombycis* diagnosis by small subunit ribosomal RNA (SSU rRNA) gene. Indian J. Agric. Res..

[92] Esvaran V., Jagadish A., Terenius O., Suraporn S., Mishra R.K., Ponnuvel K.M. (2020). Targeting essential genes of *Nosema* for the diagnosis of pebrine disease in silkworms. Ann. Parasitol..

[93] Wu Y.-X., Sadiq S., Jiao X.-H., Zhou X.-M., Wang L.-L., Xie X.-R., Khan I., Wu P. (2024). CRISPR/Cas13a-mediated visual detection: A rapid and robust method for early detection of *Nosema bombycis* in silkworms. Insect Biochem. Mol. Biol..

[94] Esvaran V.G., Gupta T., Nayaka A.R.N., Sivaprasad V., Ponnuvel K.M. (2018). Molecular characterization of *Nosema bombycis* methionine aminopeptidase 2 (*MetAP2*) gene and evaluation of anti-microsporidian activity of Fumagilin-B in silkworm *Bombyx mori*. 3 Biotech.

[95] Senderskiy I.V., Dolgikh V.V., Ismatullaeva D.A., Mirzakhodjaev B.A., Nikitina A.P., Pankratov D.L. (2024). Treatment of microsporidium *Nosema bombycis* spores with the new antiseptic M250 helps to avoid bacterial and fungal contamination of infected cultures without affecting parasite polar tube extrusion. Microorganisms.

[96] Liu H., Chen B., Hu S., Liang X., Lu X., Shao Y. (2016). Quantitative proteomic analysis of germination of *Nosema bombycis* spores under extremely alkaline conditions. Front. Microbiol..

[97] Meng X.Z., Luo B., Tang X.Y., He Q., Xiong T.R., Fang Z.Y., Pan G., Li T., Zhou Z.Y. (2018). Pathological analysis of silkworm infected by two microsporidia *Nosema bombycis* CQ1 and *Vairimorpha necatrix* BM. J. Invertebr. Pathol..

[98] Zhang X., Feng H., He J., Liang X., Zhang N., Shao Y., Zhang F., Lu X. (2022). The gut commensal bacterium *Enterococcus faecalis* LX10 contributes to defending against *Nosema bombycis* infection in *Bombyx mori*. Pest Manag. Sci..

[99] Bauer L.S., Miller D.L., Maddox J.V., McManus M.L. (1998). Interactions between a *Nosema* sp. (Microspora: Nosematidae) and nuclear polyhedrosis virus infecting the gypsy moth, *Lymantria dispar* (Lepidoptera: Lymantriidae). J. Invertebr. Pathol..

[100] Hill C., Guarner F., Reid G., Gibson G.R., Merenstein D.J., Pot B., Morelli L., Canani R.B., Flint H.J., Salminen S. (2014). Expert consensus document. The International Scientific Association for Probiotics and Prebiotics consensus statement on the scope and appropriate use of the term probiotic. Nat. Rev. Gastroenterol. Hepatol..

[101] Anee I.J., Alam S., Begum R.A., Shahjahan R.M., Khandaker A.M. (2021). The role of probiotics on animal health and nutrition. JoBAZ.

[102] Savio C., Mugo-Kamiri L., Upfold J.K. (2022). Bugs in bugs: The role of probiotics and prebiotics in maintenance of health in mass-reared insects. Insects.

[103] Vásquez A., Forsgren E., Fries I., Paxton R.J., Flaberg E., Szekely L., Olofsson T.C. (2012). Symbionts as Major Modulators of Insect Health: Lactic Acid Bacteria and Honeybees. PLoS ONE.

[104] Ramos O.Y., Basualdo M., Libonatti C., Vega M.F. (2020). Current status and application of lactic acid bacteria in animal production systems with a focus on bacteria from honey bee colonies. J. Appl. Microbiol..

[105] Barretto D.A., Gadwala M., Vootla S.K., Gurtler V., Subrahmanyam G. (2021). The silkworm gut microbiota: A potential source for biotechnological applications. Methods in Microbiology.

[106] Arasakumar E., Vasanth V., Vijay S., Swathiga G. (2023). Role of microorganisms in the gut of silkworms. J. Pharm. Innov..

[107] Singh K.K., Chauhan R.M., Pande A.B., Gokhale S.B., Hegde N.G. (2005). Effect of use of *Lactobacillus plantarum* as probiotics to improve cocoon production of mulberry silkworm, *Bombyx mori* (L.). J. Basic Appl. Sci..

[108] Esaivani C., Vasanthi K., Bharathi R., Chairman K. (2014). Impact of probiotic *Saccharomyces cerevisiae* on the enzymatic profile and the economic parameters of silkworm *Bombyx mori* L.. Adv. Biol. Biomed..

[109] Suraporn S., Sangsuk W., Chanhan P., Promma S. (2015). Effects of probiotic bacteria on the growth parameters of the Thai silkworm, *Bombyx mori*. Thai J. Agric. Sci..

[110] Taha R.H., Kamel H.M. (2017). Micro-organisms supplementation to mulberry silkworm, *Bombyx mori* L.. Egypt. Acad. J. Biol. Sci. Entomol..

[111] Mala M., Vijila K. (2018). Beneficial effects of *Bacillus licheniformis* and *Bacillus niabensis* on growth and economic characteristics of silkworm, *Bombyx mori* L.. Int. J. Chem. Stud..

[112] Saranya M., Krishnamoorthy S., Murugesh K. (2019). Fortification of mulberry leaves with indigenous probiotic bacteria on larval growth and economic traits of silkworm (*Bombyx mori*, L.). J. Entomol. Zool. Stud..

[113] Sekar P., Kalpana S., Ganga S., John G., Kannadasan N. (2016). Effect of the probionts to the enhancement of silk proteins (sericin and fribroin) in the silk gland and cocoons of silkworm (LxCSR2) *Bombyx mori* (L.). Int. J. Pharm. Biol. Sci..

[114] Gunasekhar V., Somayaji A. (2019). Effect of endophytic bacteria *Burkholderia cepacia* on growth, cocoon characters and enzyme activity of silkworm, *Bombyx mori* L.. South Asian J. Res. Microbiol..

[115] Yeruva T., Vankadara S., Ramasamy S., Lingaiah K. (2020). Identification of potential probiotics in the midgut of mulberry silkworm, *Bombyx mori*, through metagenomic approach. Probiot. Antimicrob. Proteins.

[116] Unban K., Klongklaew A., Kodchasee P., Pamueangmun P., Shetty K., Khanongnuch C. (2022). Enterococci as dominant xylose utilizing lactic acid bacteria in Eri silkworm midgut and the potential use of *Enterococcus hirae* as probiotic for Eri culture. Insects.

[117] Rajakumari D.V.S., Padmalatha C., Das S.S.M., Ranjitsingh A.J.A. (2007). Efficacy of probiotic and neutraceutical feed supplements against flacherie disease in mulberry silkworm, *Bombyx mori* L.. Indian J. Sericult..

[118] Subramanian S., Mohanrag P., Muthuswamy M. (2009). New paradigm in silkworm disease management using probiotic application of *Streptomyces noursei*. Karnataka J. Agric. Sci..

[119] Nishida S., Ishii M., Nishiyama Y., Abe S., Ono Y., Sekimizu K. (2017). *Lactobacillus paraplantarum* 11-1 isolated from rice bran pickles activated innate immunity and improved survival in a silkworm bacterial infection model. Front. Microbiol..

[120] Alcosaba D.M. (2019). Effect of fungus, *Trichoderma harzianumas* probiotic on the growth, cocoon parameters, silk characters and resistance of silkworms (*Bombyx mori*) challenged by muscardine disease-causing *Metarhizium*. Ascendens Asia J. Multidiscip. Res. Abstr..

[121] Wang F.W., Lu X.M., Huang S.K. (2003). Kinetic studies of Enterococci inhibition on the germination of *Nosema bombycis* spores. Acta Serol. Sin..

[122] Suraporn S., Terenius O. (2021). Supplementation of *Lactobacillus casei* reduces the mortality of *Bombyx mori* larvae challenged by *Nosema bombycis*. BMC Res. Notes.

[123] Khezrian A., Bagheri M., SouratiZanjani R., KheirkhahRahimabad Y., Nematollahian S., Zahmatkesh A. (2022). The effects of feed supplements and *Nosema bombycis* infection on economical traits in various silkworm breeding lines. Entomol. Exp. Appl..

[124] Georghe A., Hăbeanu M., Mihalcea T., Diniță G., Moise A.R. (2024). Probiotics Supplementation to Mulberry Silkworm *B. mori*. Sci. Pap. Anim. Sci. Biotechnol..

[125] Li G., Xiao Y., Leng J., Lou Q., Zhao T. (2024). Beneficial efficacy and mode of action of probiotic *Bacillus subtilis* SWL−19 on the silkworm (*Bombyx mori* L.). Symbiosis.

[126] Nishida S., Ono Y., Sekimizu K. (2016). Lactic acid bacteria activating innate immunity improve survival in bacterial infection model of silkworm. Drug Discov Ther..

[127] Xu S., Wu X., Wei Y., He L., Xu F. (2024). Investigating the mechanism of *Lactobacillus reuteri* FLRE589 on the growth and cultivation of silkworms (*Bombyx mori*). Symbiosis.

[128] Zeng Z., Tong X., Yang Y., Zhang Y., Deng S., Zhang G., Dai F. (2024). *Pediococcus pentosaceus* ZZ61 enhances growth performance and pathogenic resistance of silkworm *Bombyx mori* by regulating gut microbiota and metabolites. Bioresour. Technol..

[129] Chen X., Ye A., Wu X., Qu Z., Xu S., Sima Y., Wang Y., He R., Jin F., Zhan P. (2022). Combined analysis of silk synthesis and hemolymph amino acid metabolism reveal key roles for glycine in increasing silkworm silk yields. Int. J. Biol. Macromol..

[130] Li G., Wu M., Xiao Y., Tong Y., Li S., Qian H., Zhao T. (2024). Multi-omics reveals the ecological and biological functions of Enterococcus mundtii in the intestine of lepidopteran insects. Comp. Biochem. Physiol. Part D Genom. Proteom..

[131] Suraporn S., Cansee S., Hupfauf S., Klammsteiner T. (2024). Lactic Acid Bacteria from *Bombyx mori* Frass: Probiotic Properties and Antagonistic Activities. Agriculture.

[132] Liang Y., Wang T., Yang W., Chen Z., Li Q., Swevers L., Liu J. (2023). Silencing of the immune gene BmPGRP-L4 in the midgut affects the growth of silkworm (*Bombyx mori*) larvae. Insect Mol. Biol..

[133] Liu J., Yang W., Liao W., Huang Y., Chen W., Bu X., Huang S., Jiang W., Swevers L. (2024). Immunological function of *Bombyx* Toll9-2 in the silkworm (*Bombyx mori*) larval midgut: Activation by *Escherichia coli*/lipopolysaccharide and regulation of growth. Arch. Insect Biochem. Physiol..

[134] Liu J., Chen W., Situ J., Li J., Chen J., Lai M., Huang F., Li B. (2024). BmToll9-1 is a positive regulator of the immune response in the silkworm *Bombyx mori*. Insects.

[135] Yang W., Lin Y., He Y., Li Q., Chen W., Lin Q., Swevers L., Liu J. (2024). BmPGPR-L4 is a negative regulator of the humoral immune response in the silkworm *Bombyx mori*. Arch. Insect Biochem. Physiol..

[136] Feng M., Fei S., Xia J., Labropoulou V., Swevers L., Sun J. (2020). Antimicrobial peptides as potential antiviral factors in insect antiviral immune response. Front. Immunol..

[137] Xia J., Fei S., Wu H., Yang Y., Yu W., Zhang M., Guo Y., Swevers L., Sun J., Feng M. (2023). The piRNA pathway is required for nucleopolyhedrovirus replication in Lepidoptera. Insect Sci..

[138] Xia J., Peng R., Fei S., Awais M.M., Lai W., Huang Y., Wu H., Yu Y., Liang L., Swevers L. (2024). Systematic analysis of innate immune-related genes in the silkworm: Application to antiviral research. Insect Sci..

[139] Fan X., Zhang Y., Guo R., Yue K., Smagghe G., Lu Y., Wang L. (2024). Decoding epitranscriptomic regulation of viral infection: Mapping of RNA N^6^-methyladenosine by advanced sequencing technologies. Cell Mol. Biol. Lett..

[140] Ren F., Yan J., Wang X., Xie Y., Guo N., Swevers L., Sun J. (2023). Peptidoglycan recognition protein S5 of *Bombyx mori* facilitates the proliferation of *Bombyx mori* Cypovirus 1. J. Agric. Food Chem..

